# Foot Dysfunction and Its Associations with Kinesiophobia and Concern About Falling in Adults with Fibromyalgia Syndrome: A Cross-Sectional Study

**DOI:** 10.3390/jcm15197718

**Published:** 2026-10-07

**Authors:** Coral Moya-Cuenca, Ana López-González, Hayla Pérez-Matilla, Alba Gracia-Sánchez, África Lozano-Espinosa, Silvia García-Lázaro, Esther Chicharro-Luna, Sara Zúnica-García

**Affiliations:** 1 Departamento de Ciencias del Comportamiento y Salud, Área Enfermería, Facultad de Medicina, Universidad Miguel Hernández, 03550 Alicante, Spain; cmoya@umh.es (C.M.-C.); analg152003@gmail.com (A.L.-G.); haylaperez@gmail.com (H.P.-M.); agracia@umh.es (A.G.-S.); szunica@umh.es (S.Z.-G.); 2 University Podiatry Clinic, Universidad Miguel Hernández, 03550 Alicante, Spain; africa.lozanoe@umh.es (Á.L.-E.); silvia.garcial@umh.es (S.G.-L.); 3 Institute of Health and Biomedical Research of Alicante (ISABIAL), 03010 Alicante, Spain

**Keywords:** fibromyalgia syndrome, foot function, kinesiophobia, concern about falling, Tampa Scale for Kinesiophobia-11, Falls Efficacy Scale–International

## Abstract

**Background/Objectives:** Foot problems are common in fibromyalgia syndrome (FMS) and may be related to pain, kinesiophobia, and concern about falling. This study examined associations between foot function, kinesiophobia, and concern about falling in adults with FMS and identified factors independently associated with worse foot function. **Methods:** Adults with clinically diagnosed FMS were assessed between December 2024 and April 2025 in this cross-sectional study. Foot function was evaluated using the Foot Function Index (FFI), kinesiophobia with the Tampa Scale for Kinesiophobia-11 (TSK-11), and concern about falling with the Falls Efficacy Scale–International (FES-I). Multivariable linear regression identified factors independently associated with total FFI score. **Results:** Among 444 participants, 95.3% were women. The mean FFI score was 52.28 ± 23.24/100, with pain showing the greatest impairment. The mean TSK-11 and FES-I scores were 29.21 ± 7.91 and 39.73 ± 12.41, respectively. FFI scores were associated with both TSK-11 and FES-I scores in bivariate analyses (*p* < 0.001). In multivariable analysis (adjusted R^2^ = 0.366), older age was associated with lower FFI scores, whereas higher BMI, smoking history, rheumatoid arthritis, and higher FES-I scores were independently associated with worse foot function. **Conclusions:** Foot dysfunction in FMS was associated with kinesiophobia and concern about falling, but only concern about falling remained independently associated after adjustment. Higher BMI, smoking history, and rheumatoid arthritis were also associated with worse foot function. Assessment of concern about falling may provide complementary information when evaluating foot-related functional impairment in individuals with FMS.

## 1. Introduction

Fibromyalgia syndrome (FMS) is a chronic condition characterized by widespread musculoskeletal pain, persistent fatigue, sleep disturbances, cognitive impairments, and affective symptoms such as anxiety and depression [1]. FMS substantially affects physical functioning [2] and quality of life [3]. Globally, it affects approximately 2–6% of the population, with a markedly higher prevalence in women [4]. In Spain, prevalence has been estimated at 2.45%, with higher rates among women, individuals with obesity, and those aged 60–69 years [5].

In the present study, foot dysfunction refers to foot-related pain, disability, and activity limitation. Functional impairment in FMS frequently involves mobility and the lower extremities. Recent evidence has shown that higher levels of pain and fatigue are associated with poorer performance on fast mobility tests in individuals with FMS [6]. Within this broader functional impairment, foot function may be particularly affected, with patients reporting foot pain, disability, and activity limitation [7,8]. Structural and clinical alterations described in this population include joint stiffness, hallux degeneration [9], plantar trigger points [8,10], peripheral neuropathies [11,12], and postural abnormalities such as pronated foot posture, especially in individuals with joint hypermobility [13]. These alterations may further compromise gait, mobility, and the ability to perform daily activities. Assessing foot-specific impairment may therefore provide information beyond general functional assessment by identifying pain and mobility limitations specifically related to the foot.

Beyond pain and structural alterations, behavioral and psychological factors may also influence functional limitation. Kinesiophobia, defined as an excessive fear of movement related to anticipated pain or reinjury, is common in chronic pain populations [14,15,16] and has also been reported in individuals with FMS [17,18,19,20]. In this population, higher levels of kinesiophobia have been associated with physical inactivity, fatigue, and greater functional impairment [21]. In the context of chronic musculoskeletal pain, pain-related fear may lead to avoidance or modification of movement, physical inactivity, and guarded movement, which may contribute to disability [22,23].

Concern about falling may represent another relevant factor affecting mobility and daily functioning in FMS. Although kinesiophobia and concern about falling are both fear-related constructs, they are conceptually distinct: kinesiophobia primarily refers to fear of movement associated with the anticipation of pain or injury, whereas concern about falling reflects concern about the possibility of falling during daily activities. Previous studies have reported an association between kinesiophobia and fear of falling in this population [24], suggesting that fear-related responses may interact with physical limitations and contribute to activity restriction. Examining both constructs simultaneously may help determine their respective associations with foot-specific disability. However, although kinesiophobia and concern about falling have been studied in relation to general functional impairment and mobility in FMS, their respective associations with foot-specific disability, particularly when considered simultaneously, remain insufficiently understood.

Therefore, this study aimed to examine the associations between foot function, kinesiophobia, and concern about falling in adults with FMS, and to identify factors independently associated with worse foot function. We hypothesized that higher levels of kinesiophobia and concern about falling would be associated with greater foot-related functional impairment.

## 2. Materials and Methods

### 2.1. Study Design

A cross-sectional observational study was conducted between December 2024 and April 2025 in accordance with the STROBE guidelines [25]. Ethical approval was granted by the Research Ethics Committee (CEI-SP; code 20241129/06/P).

### 2.2. Participants

Participants were recruited through seven clinical and patient-association settings located in the provinces of Alicante and Murcia, Spain: the Fibromyalgia Unit of San Vicente del Raspeig Hospital (Alicante), Asociación de Fibromialgia de Elda-Petrer Comarca (ASFIEL), Asociación de Fibromialgia de Villena y Comarca (AFIVIC), Asociación de Salud Integral y Mejoras Psicofísicas y Psicosociales (ASIMEPP), Asociación de Fibromialgia y Síndrome de Fatiga Crónica de Calpe, Asociación Ilicitana de Fibromialgia (AIFI), and Asociación de Fibromialgia, Fatiga Crónica y otras Enfermedades Reumáticas y Músculo-Esqueléticas de Murcia (FIBROFAMUR).

The participating centers disseminated information about the study among their patients or members. Individuals interested in participating were initially screened by a healthcare professional at each center to verify the eligibility criteria. A previous diagnosis of FMS established by a rheumatologist was confirmed through clinical documentation available at the participating center. Eligible participants were subsequently assessed in person, and the questionnaires were administered through face-to-face interviews.

Inclusion criteria were: a previous clinical diagnosis of FMS established by a rheumatologist and confirmed through clinical documentation, age ≥ 18 years, ability to ambulate at assessment, and written informed consent. The specific diagnostic criteria and version/year used by the diagnosing rheumatologist were not systematically recorded. Exclusion criteria were: cognitive impairment or severe mental disorder precluding questionnaire completion, and history of lower-limb musculoskeletal surgery or injury within the previous six months.

### 2.3. Size Calculation

The original sample-size calculation was based on the precision required to estimate the FFI score. Sample size was estimated using software from the Clinical Epidemiology and Biostatistics Unit of the University Hospital Complex of A Coruña (www.fisterra.com). Assuming a 95% confidence level, a precision of 3 points, and an SD of 20.39 points on the FFI [9], the minimum sample of 178 participants was required. Allowing for 15% potential non-response or incomplete data, the target sample was 209. This sample size calculation was intended to estimate the mean total FFI score with a specified precision and was not specifically designed for hypothesis testing or multivariable regression analysis.

### 2.4. Data Collection

*Sociodemographic variables*

Recorded sex (female/male), age (years), educational level (none/primary; secondary; university), marital status (single; married; divorced/separated; widowed), and employment status (employed; unemployed; on medical leave; retired/pensioner). Education, marital status, and employment were treated as categorical variables as specified.

*Anthropometrics*

Weight (kg) and height (cm) were measured during the in-person assessment, and body mass index (BMI) was calculated as weight/height^2^ (kg/m^2^). BMI categories followed the World Health Organization (WHO) criteria.

*Lifestyle factors*

Smoking status was categorized as current, former, or never smoker based on participant report. Physical activity was assessed during the face-to-face interview by asking participants about the type and frequency of physical activity usually performed. Based on this information, weekly physical activity was estimated and dichotomized according to WHO recommendations: participants meeting ≥ 150 min/week of moderate-intensity activity were classified as physically active; otherwise, inactive.

*Clinical variables related to FMS*

We recorded years since FMS diagnosis, years since first symptom, diagnostic latency (computed as years from first symptom to formal diagnosis), age at diagnosis, family history of FMS, and the presence of comorbidities. Comorbidities were identified from clinical documentation provided by the participants and were recorded as present or absent.

*Foot function*

To assess foot-related pain, disability, and activity limitation, the Foot Function Index (FFI) was administered. This self-administered questionnaire comprises 23 items across three subscales: pain (9 items), disability (9 items), and activity limitation (5 items). Each item is scored on a visual analog scale from 0 to 9 (0 = lowest, 9 = highest). Items not applicable to the participant were left unanswered. The two pain items related to insoles were completed only by current insoles users; responses from non-users were considered non-applicable rather than missing data. The total FFI score was calculated as the sum of answered items divided by the maximum possible score for those items (207 if none were missing), multiplied by 100, and expressed on a 0–100 scale; higher scores indicate greater impairment of foot function [26,27].

*Kinesiophobia*

Kinesiophobia was assessed using the Spanish version of the Tampa Scale for Kinesiophobia-11 (TSK-11), a widely used instrument in chronic pain populations. The questionnaire consists of 11 items scored on a 4-point Likert scale (1 = strongly disagree; 4 = strongly agree). Lower scores indicate less kinesiophobia, whereas higher scores reflect greater fear of movement [28].

*Concern about falling*

Concern about falling was evaluated using the Spanish version of the Falls Efficacy Scale–International (FES-I). This scale includes 16 items; each is rated from 1 (“not at all concerned”) to 4 (“very concerned”). The total score ranges from 16 to 64, with higher scores indicating greater concern about falling [29]. The FES-I has previously been used to assess concern about falling in individuals with FMS [30].

### 2.5. Use of Generative Artificial Intelligence

Generative artificial intelligence (GenAI) was used solely to assist in the preparation of the graphical abstract. The final graphical abstract was reviewed and approved by the authors, who take full responsibility for its content and accuracy.

### 2.6. Statistical Analysis

Data were analyzed using IBM SPSS Statistics software, version 29 (IBM Corp., Armonk, NY, USA). Qualitative variables were described using frequency distributions (counts and percentages), while quantitative variables were presented as mean and standard deviation. The normality of variables was assessed using the Kolmogorov–Smirnov test.

For comparisons of quantitative variables between two groups, the non-parametric Mann–Whitney U test was used, and for comparisons among more than two groups, the Kruskal–Wallis test was applied. Correlations between quantitative variables were analyzed using Spearman’s correlation coefficient.

Additionally, a multivariable linear regression analysis was performed with the total FFI score as the dependent variable. TSK-11 and FES-I total scores were considered the main variables of interest. A fixed set of clinically relevant covariates was included in all models: age, sex, BMI, smoking status, physical activity, and rheumatoid arthritis. BMI was entered as a continuous variable; smoking status was dichotomized as never smoker versus ever smoker (current or former smoker); physical activity was coded as active versus inactive; and rheumatoid arthritis as absent versus present. Three models were fitted using the enter method: Model 1 included TSK-11 and the adjustment covariates; Model 2 included FES-I and the same covariates; and Model 3 included both TSK-11 and FES-I together with the same adjustment set. Multicollinearity was evaluated using tolerance and variance inflation factor (VIF) values. Linearity, homoscedasticity, and residual normality were assessed by visual inspection of residual plots, and influential observations were evaluated using Cook’s distance. Regression coefficients, 95% CI, R^2^, adjusted R^2^, and overall F statistics were reported. A sensitivity analysis was also performed by repeating the joint model after excluding participants with rheumatoid arthritis. A significance level of *p* < 0.05 was established.

## 3. Results

A total of 444 individuals with FMS were included; 4.7% (n = 21) were men and 95.3% (n = 423) were women. The mean age was 55.05 ± 9.48 years (95% CI: 54.17–55.94), and the mean BMI was 27.22 ± 5.33 kg/m^2^ (95% CI: 26.72–27.72).

The mean time since FMS diagnosis was 11.88 ± 10.09 years. A family history of FMS was reported by 25.7% (n = 114). The most frequent comorbidities were anxiety (70.5%), depression (60.1%), migraine (45.9%), and disk hernia (42.3%) (Table 1).

With respect to podiatric care, 68.5% (n = 304) had previously received podiatric treatment. Overall, 52.3% (n = 232) had undergone chiropody and 32.3% (n = 143) had used plantar orthoses; 25.2% (n = 112) were current orthoses users at the time of assessment.

The mean total FFI score was 52.28 ± 23.24 (95% CI: 50.11–54.44). The pain subscale was the most affected, with a mean of 63.10 ± 25.64 (95% CI: 60.71–65.49). Within this subscale, the highest-scoring item was “pain at the end of the day” (6.43 ± 2.54; 95% CI: 6.19–6.66) (Table 2).

The mean TSK-11 score was 29.21 ± 7.91 (95% CI: 28.47–29.95), and the mean FES-I score was 39.73 ± 12.41 (95% CI: 38.58–40.89).

Men showed higher TSK-11 scores than women (33.33 ± 6.07 vs. 29.01 ± 7.91; *p* = 0.014). The Hodges–Lehmann estimated difference was 4 points (95% CI: 1 to 8). Significant overall differences according to educational level were observed for FFI (*p* = 0.001), TSK-11 (*p* < 0.001), and FES-I (*p* < 0.001). Significant overall differences according to smoking status were observed for FFI (*p* = 0.023) and TSK-11 (*p* = 0.034). Physical inactivity was associated with worse foot function, kinesiophobia, and concern about falling (all *p* < 0.001) (Table 3).

All FFI subscales showed significant correlations with kinesiophobia (TSK-11) and concern about falling (FES-I) (all *p* < 0.001). The strongest correlation was between the FFI disability subscale and FES-I (r_s_ = 0.608) (Table 4).

Finally, in the separate adjusted models, both TSK-11 and FES-I were independently associated with total FFI score. In the joint model, FES-I remained independently associated with FFI (B = 0.997, 95% CI 0.817 to 1.176; *p* < 0.001), whereas the association with TSK-11 was attenuated and was no longer statistically significant (B = 0.137, 95% CI −0.145 to 0.420; *p* = 0.340). Age, BMI, smoking history, and rheumatoid arthritis were also independently associated with FFI. The joint model was statistically significant (R^2^ = 0.377; adjusted R^2^ = 0.366; F (8.435) = 32.929; *p* < 0.001; n = 444) (Table 5). Detailed results for the separate models and the sensitivity analysis excluding participants with rheumatoid arthritis are provided in the Supplementary Materials.

## 4. Discussion

The present study examined the relationships between foot function, kinesiophobia, and concern about falling in individuals with FMS. Foot-related impairment was substantial in this sample, with pain representing the most affected FFI domain. Both kinesiophobia and concern about falling were significantly associated with poorer foot function in the bivariate analyses. However, after multivariable adjustment, concern about falling remained independently associated with total FFI score, whereas the association with kinesiophobia was attenuated. Higher BMI, smoking history, and rheumatoid arthritis were also independently associated with worse foot function. These findings support the view that foot-related disability may represent a clinically relevant manifestation of the broader multidimensional functional impairment experienced by individuals with FMS, particularly in relation to mobility and concern about falling.

Foot-related impairment was notable in the present sample, with pain representing the most affected FFI domain. This finding is consistent with previous studies reporting substantial foot pain, disability, and activity limitation in individuals with FMS [7,8,9,10,11]. Ciaffi et al. [7] described a close relationship between FMS severity and foot-related problems assessed using the FFI. Together, these findings support the clinical relevance of specifically assessing foot-related symptoms and functional limitations in FMS rather than considering lower-limb impairment solely as part of generalized pain.

Kinesiophobia was significantly associated with poorer foot function in the bivariate analysis, in line with previous studies reporting relationships between kinesiophobia and disability, reduced physical activity, and poorer functional performance in individuals with FMS [20,21,22,31,32,33]. However, this association was attenuated after multivariable adjustment, and TSK-11 did not remain independently associated with total FFI score. This suggests that the relationship between kinesiophobia and foot-related impairment may overlap with other clinical and psychological factors. Kinesiophobia may therefore reflect a broader fear-related dimension of functional limitation in FMS rather than an independent correlate of foot dysfunction.

Regarding the magnitude of kinesiophobia, the mean TSK-11 score observed in the present study (29.21 ± 7.91) was higher than that reported by Cigarán-Méndez et al. [34] (24.9 ± 7.5) and León-Llamas et al. [24] (25.68 ± 6.54) in individuals with FMS, and was very similar to that reported by Peinado-Rubia et al. [35] (28.88 ± 7.11). These differences may partly reflect variations in sample characteristics across studies. Previous studies using the original 17-item TSK have also reported higher kinesiophobia scores in individuals with FMS than in healthy controls [20,36]. However, because the TSK-17 and TSK-11 have different score ranges, direct numerical comparisons between these instruments are not appropriate.

Concern about falling showed a particularly strong relationship with foot function, especially with the disability domain of the FFI. Moreover, FES-I remained strongly associated with total FFI score in the multivariable model. However, this association should be interpreted cautiously because both FFI and FES-I are self-reported instruments and include constructs related to mobility and perceived functional difficulty. Therefore, part of the observed association may reflect construct overlap and common-method variance rather than an entirely independent clinical relationship. Previous studies have reported associations between concern about falling and poorer physical performance, mobility, and perceived functional capacity in this population [37,38]. Although the present cross-sectional data cannot establish directionality, the observed relationship is compatible with the possibility that foot-related disability and concern about falling form part of a broader pattern of mobility limitation in FMS.

The different behavior of TSK-11 and FES-I in the adjusted model is particularly noteworthy. TSK-11 remained significantly associated with FFI after adjustment for the prespecified clinical covariates when FES-I was not included in the model. However, its coefficient was markedly attenuated after FES-I was added to the joint model, whereas FES-I remained strongly associated with FFI. This pattern suggests substantial overlap between the two fear-related constructs and their relationship with perceived functional impairment, rather than indicating that kinesiophobia is unrelated to foot dysfunction. The moderate correlation observed between TSK-11 and FES-I further supports this interpretation. Longitudinal studies are needed to clarify the temporal and potentially distinct contributions of kinesiophobia and concern about falling to foot-related impairment in FMS.

BMI was also independently associated with poorer foot function. Previous research has described a high prevalence of overweight and obesity in FMS and an association between higher BMI and greater disease burden [39]. Excess body mass may also be associated with altered lower-limb biomechanics and increased mechanical loading of foot structures [9,40,41,42]. However, given the cross-sectional nature of the present study, these findings should be interpreted as associations rather than evidence of a causal effect of BMI on foot dysfunction.

Smoking history was independently associated with higher FFI scores. However, this finding should be interpreted cautiously because smoking was dichotomized as ever versus never smoking, thereby combining current and former smokers and without accounting for smoking intensity, duration, or cumulative exposure. Consequently, exposure misclassification and residual confounding cannot be excluded. Smoking may also coexist with other clinical or behavioral characteristics that were not fully captured in the present study. Therefore, this association should not be interpreted as evidence of a causal effect of smoking on foot dysfunction. Rheumatoid arthritis was also independently associated with worse foot function. This finding is clinically plausible, as foot and ankle involvement in rheumatic diseases, particularly rheumatoid arthritis, has been associated with pain, mobility restrictions, and activity limitations [43]. However, the mechanisms underlying this association cannot be determined from the present study, and residual confounding cannot be excluded.

An inverse association between age and total FFI score was also observed after adjustment, indicating slightly lower FFI scores with increasing age. Given the small effect magnitude of this association and the cross-sectional design, this finding should not be interpreted as evidence that older age is protective against foot dysfunction. Possible explanations for this unexpected finding include differences in activity levels, adaptation to long-standing symptoms, or other unmeasured factors. These explanations are speculative and cannot be confirmed from the present cross-sectional data.

From a clinical perspective, these findings suggest that assessment of individuals with FMS who report foot problems may benefit from extending beyond pain intensity alone. Incorporating measures of foot-specific function and concern about falling may provide additional information about mobility-related limitations. The FFI and FES-I may therefore offer complementary perspectives on functional impairment, while TSK-11 may help characterize fear-related responses even though it did not retain an independent association with FFI after adjustment. Whether interventions targeting these factors improve foot-related outcomes cannot be determined from the present study and should be examined prospectively.

Overall, these findings support a broader functional assessment in FMS that considers not only pain, but also foot-specific disability and concern about falling, while acknowledging that the cross-sectional design precludes causal or temporal inference.

This study has several strengths, including the large sample size, the use of validated measurement instruments, and the inclusion of multivariable analysis to examine factors independently associated with foot function. Participants comprised a large sample of adults with rheumatologist-confirmed FMS recruited through a hospital unit and patient associations in Alicante and Murcia. The use of the FFI enabled a specific assessment of foot-related pain, disability, and activity limitation, an area that remains relatively underexplored in FMS. In addition, the joint assessment of foot function, kinesiophobia, and concern about falling provides further insight into an underexplored aspect of functional impairment in FMS.

Several limitations should also be acknowledged. First, the cross-sectional design precludes causal or temporal inferences. Second, the sample was predominantly female, reflecting the epidemiology of FMS; however, sex-based comparisons should be interpreted cautiously because of the marked imbalance between men and women, particularly the small number of male participants. The bivariate analyses involved multiple comparisons without adjustment for multiplicity and should therefore be regarded as exploratory. Third, recruitment through patient associations may have introduced selection bias, as individuals affiliated with these organizations may differ from the broader population with FMS in disease severity, healthcare engagement, or other characteristics. Reliance on self-reported questionnaires may also introduce response and common-method bias. In particular, because both the FFI and FES-I are self-reported measures that include aspects of perceived mobility and functional difficulty, part of the observed association between these instruments may reflect construct overlap and common-method variance rather than an entirely independent clinical relationship. Smoking exposure was assessed only as ever versus never smoking, thereby combining current and former smokers, without information on smoking intensity, duration, or cumulative exposure. This may have introduced exposure misclassification and residual confounding, and the observed association between smoking history and foot function should therefore not be interpreted causally. No objective clinical or biomechanical foot measures, such as plantar-pressure assessment, instrumented balance testing, or gait analysis, were collected. Acute pain intensity at the time of assessment was not controlled and may have influenced self-reported functional impairment. Residual confounding from unmeasured clinical, psychological, or behavioral factors cannot be excluded. Finally, the absence of a healthy control group prevents direct comparison with individuals without FMS.

## 5. Conclusions

Individuals with FMS showed substantial foot-related impairment, with pain being the most affected FFI domain. Kinesiophobia was also notable in the sample and was associated with poorer foot function, although only concern about falling remained independently associated with FFI after adjustment. Longitudinal studies are warranted to clarify these relationships and their potential clinical relevance.

## Figures and Tables

**Table 1:** Sociodemographic, clinical, and lifestyle characteristics of individuals with FMS.

Characteristics	n = 444 n (%)
**Sex**	
Male	21 (4.7)
Female	423 (95.3)
**BMI** (kg/m^2^)	
Underweight (BMI < 18.5)	7 (1.6)
Normal weight (BMI 18.5–24.9)	139 (31.3)
Overweight (BMI 25.0–29.9)	168 (37.8)
Obesity (BMI ≥ 30)	130 (29.3)
**Educational level**	
No formal education	9 (2)
Primary education	117 (26.4)
Secondary education	200 (45)
University education	118 (26.6)
**Marital status**	
Single	69 (15.5)
Married	269 (60.6)
Separated or divorced	81 (18.2)
Widowed	25 (5.6)
**Living arrangement**	
Lives alone	73 (16.4)
Lives with others	371 (83.6)
**Smoking status**	
Current smoker	101 (22.7)
Former smoker	121 (27.3)
Never smoker	222 (50)
**Physical activity level**	
Physically active	267 (60.1)
Physically inactive	177 (39.9)
**Comorbidity**	
Anxiety	313 (70.5)
Depression	267 (60.1)
Migraine	204 (45.9)
Disk hernia	188 (42.3)
Irritable bowel syndrome	148 (33.3)
Hypercholesterolemia	164 (36.9)
Hypertension	98 (22.1)
Hypothyroidism	95 (21.4)
Sleep apnea	73 (16.4)
Rheumatoid arthritis	70 (15.8)
Diabetes mellitus	30 (6.8)

**Abbreviations:** BMI, Body Mass Index; FMS, Fibromyalgia Syndrome.

**Table 2:** FFI scores in individuals with FMS.

FFI Item	Mean ± SD	95% CI
**Number of days with foot pain during the past week** (n = 444)	4.73 ± 2.46	4.50–4.96
**Pain Scale**	**63.10 ± 25.64**	**60.71–65.49**
Intensity of maximum foot pain (n = 444)	5.50 ± 2.53	5.26–5.73
Morning foot pain (n = 444)	4.68 ± 2.94	4.41–4.96
Foot pain while walking (n = 444)	5.58 ± 2.65	5.34–5.83
Foot pain while standing (n = 444)	5.81 ± 2.61	5.57–6.05
Foot pain when walking with shoes (n = 444)	5.95 ± 2.62	5.71–6.19
Foot pain when standing with shoes (n = 444)	6.18 ± 2.54	5.94–6.41
Foot pain when walking with insoles (n = 112)	4.75 ± 2.74	4.24–5.26
Foot pain when standing with insoles (n = 112)	4.85 ± 2.75	4.33–5.36
Level of foot pain at the end of the day	6.43 ± 2.54	6.19–6.66
**Disability Scale**	**57.64 ± 27.79**	**55.05–60.23**
Difficulty walking indoors (n = 444)	3.57 ± 2.84	3.31–3.83
Difficulty walking outdoors (n = 444)	4.94 ± 2.87	4.67–5.21
Difficulty walking 500 m (n = 444)	4.54 ± 3.04	4.26–4.83
Difficulty climbing stairs (n = 444)	5.97 ± 2.79	5.71–6.23
Difficulty descending stairs (n = 444)	5.66 ± 2.85	5.39–5.92
Difficulty standing on tiptoes (n = 444)	6.07 ± 2.90	5.80–6.34
Difficulty rising from a chair (n = 444)	5.28 ± 2.95	5.00–5.55
Difficulty stepping onto a curb (n = 444)	4.53 ± 2.98	4.25–4.81
Difficulty walking at a fast pace (n = 444)	6.10 ± 2.80	5.84–6.36
**Activity Limitation Scale**	**27.02 ± 25.89**	**24.61–29.43**
Staying at home all day due to foot problems (n = 444)	2.93 ± 3.03	2.65–3.21
Staying in bed all day due to foot problems (n = 444)	1.72 ± 2.57	1.48–1.96
Limiting activities due to foot problems (n = 444)	3.94 ± 3.09	3.66–4.23
Using an assistive device indoors (n = 444)	1.40 ± 2.74	1.15–1.66
Using an assistive device outdoors (n = 444)	2.18 ± 3.39	1.87–2.50
**Total FFI score**	**52.28 ± 23.24**	**50.11–54.44**

**Abbreviations:** CI, Confidence Interval; FFI, Foot Function Index; FMS, Fibromyalgia Syndrome; SD, Standard Deviation For all FFI items, n = 444, except for the two items related to insoles, which were applicable only to current insoles users (n = 112). Non-applicable responses were not considered missing data.

**Table 3:** Relationship between social, lifestyle, and clinical characteristics and scores on FFI, TSK-11, and FES-I in individuals with FMS.

	Total FFI Score	*p* Value	Total TSK-11 Score	*p* Value	Total FES-I Score	*p* Value
	Mean ± SD R r_s_.		Mean ± SD r_s_.		Mean ± SD r_s_.	
**Sex**		0.877 ^a^		0.014 ^a^ *		0.171 ^a^
Male (n = 21)	50.55 ± 25.87		33.33 ± 6.07		43.10 ± 11.46	
Female (n = 423)	52.36 ± 23.16		29.01 ± 7.91		39.57 ± 12.45	
**Age** (years)	−0.082	0.086 ^b^	0.002	0.973 ^b^	−0.027	0.564 ^b^
**BMI** (kg/m^2^)	0.164	<0.001 ^b^ *	0.147	0.002 ^b^ *	0.134	0.005 ^b^ *
**Educational level**		0.001 ^c^ *		<0.001 ^c^ *		<0.001 ^c^ *
No formal education (n = 9)	55.03 ± 28.89		33.44 ± 6.73		41.33 ± 12.25	
Primary education (n = 117)	54.23 ± 21.99		30.66 ± 7.88		40.54 ± 12.25	
Secondary education (n = 200)	55.16 ± 23.79		29.75 ± 7.73		41.52 ± 12.49	
University education (n = 118)	45.24 ± 21.86		26.55 ± 7.71		35.79 ± 11.89	
**Marital status**		0.493 ^c^		0.080 ^c^		0.696 ^c^
Single (n = 69)	54.30 ± 19.16		29.22 ± 7.55		38.72 ± 12.95	
Married (n = 269)	51.06 ± 23.20		28.71 ± 7.80		39.54 ± 12.06	
Separated or divorced (n = 81)	53.05 ± 25.78		29.83 ± 8.44		40.91 ± 12.88	
Widowed (n = 25)	57.29 ± 25.45		32.56 ± 7.74		40.80 ± 13.51	
**Living arrangement**		0.385 ^a^		0.859 ^a^		0.592 ^a^
Lives alone (n = 73)	54.59 ± 23.12		29.23 ± 8.03		40.33 ± 12.44	
Lives with others (n = 371)	51.82 ± 23.27		29.21 ± 7.90		39.62 ± 12.42	
**Smoking status**		0.023 ^c^ *		0.034 ^c^ *		0.107 ^c^
Current smoker (n = 101)	56.67 ± 23.78		31.02 ± 7.93		41.79 ± 12.66	
Former smoker (n = 121)	53.36 ± 23.08		28.66 ± 7.99		38.38 ± 12.44	
Never smoker (n = 222)	49.68 ± 22.83		28.69 ± 7.77		39.54 ± 12.22	
**Physical activity level**		<0.001 ^a^ *		<0.001 ^a^ *		<0.001 ^a^ *
Physically active (n = 267)	49.31 ± 22.79		27.86 ± 7.76		37.39 ± 11.55	
Physically inactive (n = 177)	56.75 ± 23.27		31.25 ± 7.71		43.27 ± 12.85	

**Abbreviations:** BMI, Body Mass Index; FES-I, Falls Efficacy Scale–International; FFI, Foot Function Index; FMS, Fibromyalgia Syndrome; SD, Standard Deviation; TSK-11: Tampa Scale for Kinesiophobia–11 items. ^a^ Mann–Whitney U test. ^b^ Spearman’s correlation. ^c^ Kruskal–Wallis test. * *p* < 0.05 considered statistically significant.

**Table 4:** Correlation between foot function (FFI), kinesiophobia (TSK-11), and concern about falling (FES-I) in individuals with FMS.

FFI	Total TSK-11 Score	*p* Value	Total FES-I Score	*p* Value
Pain	0.274	<0.001 *	0.372	<0.001 *
Disability	0.415	<0.001 *	0.608	<0.001 *
Activity limitation	0.348	<0.001 *	0.576	<0.001 *
Total FFI score	0.392	<0.001 *	0.590	<0.001 *

**Abbreviations:** FES-I, Falls Efficacy Scale–International; FFI, Foot Function Index; FMS, Fibromyalgia Syndrome; TSK-11, Tampa Scale for Kinesiophobia–11 items. Spearman’s correlation. * *p* < 0.05 considered statistically significant.

**Table 5:** Multivariable linear regression analysis for total FFI score in individuals with FMS.

Model	Coefficients (Unstandardized)		Coefficients (Standardized)	95% CI for B		*p* Value	VIF
	B	SE	β	Lower Bound	Upper Bound		
FES-I	0.997	0.091	0.532	0.817	1.176	<0.001 *	1.661
TSK-11	0.137	0.144	0.047	−0.145	0.420	0.340	1.668
Sex	7.369	4.221	0.067	−0.927	15.665	0.082	1.040
Age	−0.221	0.095	−0.090	−0.408	−0.035	0.020 *	1.049
BMI	0.507	0.169	0.116	0.175	0.838	0.003 *	1.048
History of smoking	4.894	1.770	0.105	1.414	8.374	0.006 *	1.016
Physical activity	0.374	1.892	0.008	−3.344	4.093	0.843	1.112
Rheumatoid arthritis	5.501	2.443	0.086	0.699	10.304	0.025 *	1.027

**Abbreviations:** BMI, Body Mass Index; CI, Confidence Interval; FES-I, Falls Efficacy Scale–International; FFI, Foot Function Index; FMS, Fibromyalgia Syndrome; SE, Standard Error; TSK-11, Tampa Scale for Kinesiophobia-11; VIF, Variance Inflation Factor. Dependent Variable: total FFI score. * *p* < 0.05 considered statistically significant.

## Data Availability

The data presented in this study are available from the corresponding author upon request because they are being used in another study.

## Supplementary Materials

The following supporting information can be downloaded at: https://www.mdpi.com/article/10.3390/jcm15197718/s1, STROBE Statement—checklist of items that should be included in reports of observational studies; Table S1: Multivariable linear regression model including TSK-11 and adjustment covariates for total FFI score; Table S2: Multivariable linear regression model including FES-I and adjustment covariates for total FFI score; Table S3: Joint multivariable linear regression model excluding participants with rheumatoid arthritis.

## Abbreviations

The following abbreviations are used in this manuscript:

FMS	Fibromyalgia Syndrome
FFI	Foot Function Index
TSK-11	Tampa Scale for Kinesiophobia-11
FES-I	Falls Efficacy Scale–International
BMI	Body Mass Index
WHO	World Health Organization
VIF	Variance inflation factor
